# Pakistani Women: Promoting Agents of Healthy Eating Habits in Catalonia—Protocol of a Culturally and Linguistically Appropriate Randomized Control Trial (RCT) Based on the Transtheoretical Model

**DOI:** 10.3390/ijerph191610386

**Published:** 2022-08-20

**Authors:** Saba Mohamed-Bibi, Jesús Contreras-Hernández, Cristina Vaqué-Crusellas

**Affiliations:** 1 Department of Social Anthropology, Faculty of Geography and History, University of Barcelona, 08001 Barcelona, Spain; 2 Observatiorio de Alimentación (ODELA), University of Barcelona, Torribera Food Campus, Santa Coloma de Gramanet, 08921 Barcelona, Spain; 3 Research Group M3O, Methodology, Methods, Models and Outcomes of Health and Social Sciences, Faculty of Health Sciences and Welfare, University of Vic-Central University of Catalonia, 08500 Vic, Spain

**Keywords:** metabolic syndrome, cardiovascular diseases, Pakistani women, South Asians, immigrant, transtheoretical model, food education

## Abstract

(1) Introduction: Dietary and lifestyle changes along with the cultural and linguistic barriers convert the immigrant women of Pakistani origin into a risk population for developing metabolic syndrome (MetS) and cardiovascular diseases (CVD). The objective of this project is to evaluate the efficacy of a culturally and linguistically appropriate food education program based on the Transtheoretical model that will allow the participants to become ambassadors of healthy eating habits for their community. (2) Methods: In this community-based RCT, any Pakistani adult woman with residence in Badalona and Santa Coloma de Gramenet will be able to participate. We will use a mixed model approach. From the quantitative perspective, the participants will answer a survey accompanied by a multilingual nutritionist that will help us to determine the sociodemographic, clinical, anthropometric, dietary data, and quality of life. From the qualitative perspective, we will conduct 6 focus groups (3 in each municipality) to determine the cultural and religious beliefs with the aim of tailoring the intervention to the target population. Hereafter, the participants from one municipality will randomly become the control group and from the other, the intervention group. The intervention group will participate in 10 weekly food education sessions based on the Transtheoretical model while the control group will receive 3 general educational sessions on food and health. During the evaluation procedure, we will assess the impact of the intervention considering the outcomes of the study. (3) Discussion: This study will establish intercultural bridges between health professionals and the Pakistani community living in Catalonia. The project will open the door for future interventions, and it will be sustainable in time as the participating women will become health promotion agents for the rest of their community.

## 1. Introduction

The number of Pakistani people living in Spain is rapidly increasing. According to the recent census data, between 2010 and 2020, the Pakistani population increased by approximately 72% [1]. Currently, 99.352 people of Pakistani origin are residing in Spain, of which 56% live in Catalonia, especially in Barcelona and its surroundings [1,2]. The group of women in this community are a minority (29%) [3] who mostly arrive in Catalonia due to the family reunification procedures predominantly sponsored by the male members of the family (husband or father) [4]. Due to cultural and linguistic barriers and difficulties in accessing the job market, immigrant women of Pakistani origin are currently one of the most invisible ethnic/racial groups in Catalonia [4]. Although the socio-demographic and socio-economic profile of the Pakistani population living in Catalonia is well known [2,3,4], the Catalan context does not have any information on its health and nutrition aspects. However, these data are available in countries with higher Pakistani populations such as Norway [5,6,7], the United Kingdom [8,9], the United States [10,11], and Australia [12].

Due to the similarity in the sociocultural and linguistic profiles [5], mostly the research in health and nutrition is conducted jointly for all the immigrant South Asian (SA) population, especially for those who proceed from India and Pakistan. It is well established that the migrant SA population tends to have a higher risk for the development of type 2 diabetes and cardiovascular disease (CVD) compared with the western population [13,14]. In such a way that the risk of developing type 2 diabetes is two to four times higher in SA as compared to Europeans [8,15,16]. They are also affected by CVD approximately a decade earlier than the Western population [17] as 25% of myocardial infarctions occur in SA under 40 years of age and more than 50% of deaths from CVD occur in SA under 50 years of age [18]. Furthermore, they have more body fat and less lean tissue than white people of European descent at any Body Mass Index (BMI) level [16,17]. SA also tends to have lower high-density lipoprotein (HDL), higher triglycerides, and increased lipoprotein (a) as compared to other ethnicities [16,17]. The prevalence of hypertension is also slightly higher in them [13]. The reunification of these factors is established as metabolic syndrome (MetS) [19]. Currently, the prevalence of MetS in SA immigrants is estimated to be 50% in the United States and 40% in the United Kingdom [19,20].

Apart from the genetic and metabolic factors, dietary and lifestyle changes caused by acculturation, account for the high prevalence of MetS in SA immigrants [5,16,17,20]. In addition to this, cultural and linguistic barriers make it difficult for them to participate in the standard health promotion programs [5,17,21]. Consequently, their health deteriorates throughout their stay in Western countries [22,23].

Different countries have successfully designed and implemented culturally and linguistically adapted health promotion programs to address this situation. A recent meta-analysis found a 35% of reduction in diabetes in SA immigrants who participate in culturally and linguistically appropriate lifestyle modification interventions [9].

In some countries, the prevalence of MetS and CVD is higher in SA women as compared to men [5,20,24]. However, this difference is more significant in immigrants of Pakistani origin [24,25]. In Norway, the risk of developing obesity and type 2 diabetes is higher in Pakistani women as compared to men [6,7,8,9,10,11,12,13,14,15,16,17,18,19,20,21,22,23,24,25]. Apart from being highly susceptible to these diseases, Pakistani women also tend to face more difficulties in integrating into the host country as compared to the men of the same origin [6,12]. They are also more affected by cultural and linguistic barriers, caused by acculturation stress and social isolation, is highly common among them [26]. Due to their physical, psychological, and socio-economic vulnerability, some countries have successfully designed and implemented culturally and linguistically appropriate food and lifestyle interventions targeting specifically immigrant women of Pakistani origin. Following this line, and taking into account all the key success factors mentioned by different authors who have conducted similar studies in other contexts [27], the current study aims to (a) evaluate the efficacy of a culturally and linguistically appropriate food education program based on the Transtheoretical Model of health behavior change [28] for Pakistani women living in Catalonia, and (b) empower the participating women to become the ambassadors of healthy eating habits in their community.

## 2. Materials and Methods

### 2.1. Study Design

The present study is community-based participatory research in which a RCT will be implemented. We will combine different methods to obtain and analyze both qualitative and quantitative data. The quantitative perspective will be used to determine the sociodemographic, health and life quality, anthropometric and dietary aspects. While the qualitative view will be used to obtain deeper information about health and nutrition beliefs and to adopt the intervention on the needs and knowledge of the target population.

### 2.2. Study Setting and Participants

This project is being carried out in the province of Barcelona, specifically in the region of Barcelonès, which is home to more than half of the foreign population of Pakistani origin residing in Catalonia [2]. Concretely, this study is taking place in Badalona and Santa Coloma de Gramenet which are two neighboring municipalities and, respectively, the second and fourth most populous in Catalonia by the Pakistani population [29].

Pakistani women living in these municipalities have the support of Fundació Ateneu Sant Roc (Badalona) and Casa Àsia (Santa Coloma de Gramenet), two institutions working with the same goal: the integration of migrant people into the host society and its environment. Both institutions accepted to participate and signed the consent form to participate in the research. Annually, Fundació Ateneu Sant Roc and Casa Asia attend between 50 to 60 Pakistani adult women. We have invited all of them and their close acquaintance to participate in the study. To facilitate the participation of Pakistani women in this community-based project we have set the inclusion and exclusion criteria as wide as possible (Table 1).

70 women associated with Casa Asia and 67 from Fundació Ateneu Sant Roc have accepted to become part of the study forming a sample of 137 women. There are three factors that indicate the homogeneity of the sample; firstly, the majority of the participants are in the process of learning Spanish and Catalan from the collaborating institutions, secondly, by belonging to the neighboring municipalities, their socioeconomic and sociodemographic conditions are similar and thirdly, all of them migrated between 2000 and 2022. So, to evade the contamination of information the participants from one institution will randomly become the control group and from the other, the intervention group.

## 3. Planned Intervention

The study will be developed in 5 different phases (dissemination and recruitment, pilot study, baseline data collection, implementation of the food education intervention and evaluation):

### 3.1. Phase 1: Dissemination and Recruitment

This phase corresponds to the dissemination of the project and the recruitment of participants for control and intervention groups. We organized introductory sessions with the educators and volunteers of the institutions, as well as their Pakistani users to present the project. Communication was realized in Urdu and Punjabi during the meetings with Pakistani women. Hereafter, the team of institutions and their users have helped us in the dissemination and recruitment procedure. We have also organized meetups at the different community spaces (social institutions, libraries, community centers, mosques, and female driving schools) to explain in-depth all the characteristics of the study to the interested women. In the end, we handed over to them the information sheets that sums up the principles of the study along with the informed consent. We also provided our contact and obtained the names, addresses, and contact numbers of those willing to participate in the project. We have completed this phase by forming a sample of 137 women.

### 3.2. Phase 2: Pilot Study

Six Pakistani women that will not be participate in the research will answer the Urdu version of the individual survey and they will also fill in the weekly food intake register. They will attend 3 general sessions about food education and they will be shown all the material of intervention in order to evaluate its cultural and linguistic appropriateness.

### 3.3. Phase 3: Baseline Data Collection

The data collection will be performed using a combination of quantitative and qualitative methods and techniques. From the quantitative perspective, the participants will answer an individual survey in Urdu guided by a multilingual nutritionist, that will collect the data about the sociodemographic, health and life quality, anthropometric and dietary aspects. In this same individual meetup, we will deliver a register about their weekly food intake and a guide on food portions specifically elaborated for the Pakistani population residing in Catalonia [30].

#### 3.3.1. Quantitative Variables

The outcome variables from the quantitative perspective are the following:

(a) Sociodemographic Data

Sociodemographic data that includes age, place of birth, marital status, academic studies, employment, languages, religion, the reason for migration, years of residence in Catalonia, and household members and their professions will be identified with the baseline survey

(b) Health Status and Quality of Life

The survey will include some questions regarding the health status of participants. They will also answer the SF12 questionnaire in the English version [31] which will help us to determine the health perception and the quality of life of participants.

(c) Nutritional Status

The nutritional status will be defined by anthropometric measurements (weight, height, and waist).

(d) Nutritional Knowledge and Culinary Skills

The survey will include an ad hoc questionnaire of 14 questions about nutritional knowledge (ability to distinguish different types of fats, familiarity with the main sources of macronutrients, knowledge about portion and frequency of consumption of different food groups, ability to read and interpret food labels and food beliefs) and 13 questions about skills (menu planning, preparing a grocery list, differentiating food groups, knowing about seasonal food, reading and interpreting food labels, preparing food with different culinary techniques, reusing food leftovers and preparing a healthy plate).

(e) Dietary Pattern

The dietary pattern will be studied through 3 instruments: (1) A survey, which will include questions about the number of daily meals, timing and place of meals, and company. (2) A Food Frequency Questionnaire (FFQ) inspired by the Table of Indicative Frequencies of the Spanish Society of Community Nutrition (SENC) [32]. (3) A weekly food record in which they indicate the type and quantity of food and drinks and the cooking methods with the help of a portion size guide specifically prepared for the participants [30] and specify the number, place, and time of the meals that they had in a week. This information will help us to assess the levels of energy consumption and the content of nutrients.

(f) Cultural and Linguistic Adequacy

At the end of the sessions, participants will answer an ad hoc satisfaction questionnaire about the linguistic, cultural, and content comprehension issues.

From the qualitative perspective, we will conduct 6 focus groups (3 in each municipality) to determine the cultural and religious beliefs related to food and the strengths and limitations of the current dietary pattern of the participants. The information obtained from the focus group will also serve us to adopt the intervention according to the sample’s needs. Their duration will be of 90 min.

#### 3.3.2. Qualitative Variables

The outcome variables from the qualitative perspective are the following:

(a) Strengths and Weaknesses of the Dietary Pattern

Through the focus groups, the participants’ traditional and current dietary patterns will be discussed. They will also describe the factors that facilitate or hinder them from following a healthy diet.

(b) Food Beliefs

The focus groups will determine the beliefs related to the consumption of different foods, their effects on health and well-being, the opinion on herbal products, the myths related to food and health, etc.

(c) Nutrition and Health Knowledge

The focus group will also help us to figure out the health and nutrition literacy of participants to adopt the intervention on their requirements.

### 3.4. Phase 4: Implementation of the Food Education Intervention

In this phase, the food education intervention based on the Transtheoretical model [28] (Table 2) will be implemented. The participants from one entity will become the control group and from the other the intervention group.

The educational sessions will be done in small groups (12–15 women) to ensure the creation of positive dynamics and to create a trustworthy environment. There will be 6 subgroups of women for the intervention group and 5 subgroups for the control group. The intervention group will participate in 10 educative sessions for 10 weeks, while the control group will attend 3 sessions. Each weekly session will have a duration of 90 min.

Due to the linguistic barriers, the sessions will be carried out in Urdu and Punjabi by a plurilingual nutritionist. All the educational material will also be translated into Urdu. The learning outcomes related to educative sessions are summarized in Table 3. During the sessions, we will emphasize the following aspects:

Encourage healthy eating habits: Bearing in mind the cultural and linguistic aspects of participants healthy eating habits will be encouraged. We will combine the healthy characteristics of their traditional diet and combine them with innovative culinary techniques to facilitate the readoption. Apart from preparing the culturally and linguistically food adapted educational guides, we will also summarize and present some local guides such as “*Petits canvis per menjar millor* (small changes to eat better)” by the Public Health Agency of Catalonia [32] to enlighten the local guidance about health and nutrition.

Implement healthy eating behaviors: To implement the healthy eating behaviors the cooking skills of participants will be worked on. They will elaborate healthy snacks and healthy plates with the traditional ingredients and culinary techniques.

Adherence to the program: To actively engage the participants in the study, the sessions will be theoretical-practical. At the end of every session, there will be a final activity. We will also conduct three practical workshops about healthy snacks, food labels, and healthy plates. Throughout the sessions, we will keep providing personal counseling to those who needed.

Empowerment: The implementation of the sessions in small groups will promote socialization and sorority among participants. We will highlight the impact of the eating habits of Pakistani women on their families and we will invite them to extend the role of healthy eating habits to the rest of the community.

### 3.5. Evaluation

The protocol study will include a process and an impact evaluation that will take part at the end of health education sessions, in which we will collect the same variables that were gathered during the baseline data collection. The only modification will be the addition of an ad hoc satisfaction questionnaire to comprehend the linguistic, cultural, and content comprehension issues. We will also repeat the 6 focus groups in order to assess the changes in food and health beliefs and nutritional knowledge and skills. Photovoice technique will also help us to determine the improvement in the eating habits, and nutrition knowledge of participants. The evaluation procedure will be repeated at 3 and 6 months.

## 4. Discussion

The number of Pakistani people in Catalonia has rapidly increased in the past 10 years. Although the data on the health and nutritional aspects of this ethnic/racial group is very limited in the Catalan context, lately, health professionals are very concerned about the increase of CVD and MetS in this community. Therefore, the population growth of this ethnic group and the rise in their health problems highlight their personalized needs in social and health aspects.

The eating habits and the beliefs about health and nutrition of Pakistani community living in Catalonia differ from the autochthonous population. Therefore, the lack of studies on aspects of health and nutrition and the absence of culturally and linguistically adapted resources are the factors that obstruct the approach, the diagnosis, and the monitoring of CVD and MetS in the immigrant population of Pakistani origin living in Catalonia. To fill this gap, we propose the first training program based on food education, in which from the designing to the evaluation we have considered all the cultural and linguistic aspects of this community, as the communication will be carried out in Urdu and Punjabi and all the material will also be translated in Urdu.

Bearing in mind the cultural aspects, we will also suggest a healthy diet that will allow the participants to keep up with their traditional eating habits and as the majority of Pakistani women living in Catalonia belong to the rural areas of Pakistan, where the gender segregation in education is a common practice, they feel uncomfortable participating in joint programs with males [4,5,6,7,8,9,10,11,12,13,14,15,16,17,18,19,20,21,22,23,24,25,26,27,28,29,30,31,32,33,34]. Apart from this, there are several reasons that led us to decide to conduct this project only for Pakistani women. Firstly, they are more vulnerable respecting the diseases mentioned above. Secondly, they are one of the most invisible ethnic groups in Catalonia as social isolation is very common among them that can cause depression and anxiety. Thirdly, the cultural and linguistic barriers affect them more profoundly. Fourthly, they are the referents in their families for food and health aspects. So, through this project, we will implement a culturally and linguistically adapted food education program based on their needs and knowledge. Participants will be trained during various sessions on nutrition and lifestyle in Urdu and Punjabi by a multilingual nutritionist.

The existing literature affirms that these types of interventions are effective to improve the nutritional and dietetic’ literacy and the food habits of this population. The fact of conducting the sessions of the program in small groups will enhance socialization and sorority among the participating women. The program will also allow the participants to become ambassadors of healthy eating habits in their households and for all the rest of the community by adopting the role of a health promotion agent. Eventually, the improvement in their health could reduce the expenditures allocated to the management of MetS and CVD.

This study will also establish intercultural bridges with health professionals. We will provide them the information about the causing factors of CVD and Mets and the culturally and linguistically adapted material to counsel their patients of Pakistani origin. The successful implementation of our project can serve as an example to create culturally adapted strategies for all the South Asian communities, especially for the Pakistani community. The health promotion agents who participated in this study will be able to contribute to the future interventions allowing them to be sustainable in the long term.

## Figures and Tables

**Table 1:** Inclusion and exclusion criteria.

**Inclusion criteria**	- Adult age (>18 years). - Immigrant women of Pakistani origin. - Residence in Badalona or Santa Coloma de Gramenet. - Voluntarily accepting to participate in the study.
**Exclusion criteria**	- Diagnosis of cognitive impairment or any physical illness that could prevent participation in the study. - Disagreement with the ethical conditions of the study.

**Table 2:** Food education intervention based on the Transtheoretical model.

Stage of Change	Objectives	Process of Change	Change Strategy
**Pre-contemplation**	Raise awareness of the problem by stimulating the possibility of change	*Consciousness raising*	Present and discuss food and lifestyle changes due to migration.
		*Dramatic relief*	Present data on the prevalence of MetS and CVD in immigrants of SA origin.
		*Self-reevaluation*	Focus group on the strengths and weaknesses of the dietary pattern.
			Debate on myths and beliefs related to food and health.
		*Environmental reevaluation*	Highlight the impact of their eating habits on future generations
**Contemplation**	Decant the scale towards change	*Consciousness raising*	Present the benefits of following healthy eating.
			Introduce the disadvantages of following an unhealthy diet.
		*Self-reevaluation*	Self-assessment of current eating habits by compiling a list of healthy and unhealthy habits.
**Preparation**	Reinforce knowledge to facilitate change	*Counterconditioning*	Conduct group sessions on food or behaviors that need to be enhanced, reduced, or changed regarding the type or quality, to follow a healthy diet.
		*Self-liberation*	Collect suggestions to acquire the recommended changes.
**Action**	Effectuate the change by promoting self-efficiency	*Stimulus or environmental control*	Workshops on dietary planning and food purchasing; elaboration of healthy dishes with traditional foods; interpretation of nutrition labeling, preparation of healthy breakfasts and snacks.
**Maintenance**	Maintain the change	*Helping relationships*	Photovoice exhibition of healthy plates.
		*Social liberation*	Acquisition of the role of health promotion agent for the rest of the Pakistani community.

**Table 3:** Food education session.

Title of the Session	Duration	Objectives	Methodology	Final Activity
**1. Why us?** **The most common health problems of the Pakistani population**	1 h	- Explain and discuss food and lifestyle changes caused by the migratory factor. - Present data on the prevalence of MetS and CVD in SA immigrants. - Present data on the eating habits of the immigrant Pakistani community.	- This session will present the most common health and nutrition problems of the migrant population of Pakistani origin, exposing their causal factors. The participants’ self-perception of health will also be discussed.	- The participants will be asked to make a list of dietary and lifestyle changes that they can make to improve their health.
**2. Food myths and beliefs: What does science say?**	1 h	- Discuss the food myths. - Demystify beliefs related to food and health. - Explain food recommendations during different stages of life.	- In this session, through a group dynamic, the food beliefs gathered during the focus groups will be discussed. Participants will express their opinion related to every myth. Eventually, all the myths will be demystified based on scientific evidence.	- At the end of the session, the worked material will be handed over in an infographic format.
**3. What is a healthy diet?**	1 h	- Define the basics of a balanced diet. - Introduce different food groups as well as macro and micronutrients. - Explain the portion and frequency of consumption of different food groups.	- In this theoretical-practical session, considering the traditional dietary pattern of the participants, the basics of healthy eating will be explained. Different food groups along with the macro and micronutrients, as well as their sources of obtention will be presented. Finally, the portion and frequency of consumption will be introduced.	- The participants will be asked to prepare a list of their healthy and unhealthy eating habits. We will also distribute a booklet with the portion and frequency of consumption, specifically elaborated for Pakistani population.
**4. The value of our traditional diet**	1 h	- Highlight the strengths and weaknesses of the traditional and actual dietary pattern of participants. - Explain the structure of a healthy plate. - Present ideas of healthy plates made with their traditional foods.	- In this session, in a group discussion, participants will reflect on the strengths and weaknesses of their traditional and actual dietary patterns. After the discussion, the structure of a healthy plate will be explained. Some examples of healthy plates elaborated with the traditional foods and cooking styles will be presented.	- As a final activity participants will draw a healthy plate. At the end of the session, a booklet with the explanation of healthy plate along with the examples worked during the sessions will be handed over.
**5. Small changes to eat better (MORE)**	1 h	- Raise awareness of the importance of daily consumption of at least 5 servings of fruit and vegetables. - Explain the benefits of eating pulses and nuts. - Propose and collect ideas to increase the consumption of fruit, vegetables, pulses, and nuts.	- From this session, we will start analyzing three blocks of a Catalan guideline “*Petits canvis per menjar millor* (small changes to eat better)”. In this first block titled “MORE” the benefits of increasing the intake of fruits, vegetables, pulses, and nuts will be presented. We will also present culturally appropriate ideas to increase the consumption of these food groups.	- Ideas and recipe suggestions will be collected to increase the consumption of fruit, vegetables, pulses, and nuts in the diet.
**6. Small changes to eat better (CHANGE)**	1.5 h	- Suggest healthy alternatives to reduce the intake of foods with low nutritional value, rich in sugar and fat. - Advice to increase the consumption of healthy, local and seasonal foods. - Prepare healthy snacks with the proposals gathered during the previous session.	- The cooking workshop will start by remembering foods with low nutritional value (soft drinks, pastries, etc.) and healthy alternatives will be proposed to replace them. Then, healthy snacks will be prepared with fresh, healthy, local and seasonal foods.	- A calendar of seasonal fruits and vegetables of Catalonia along with a recipe book based on the proposals collected during the previous session will be distributed.
**7. Small changes to eat better (LESS)**	1 h	- Warn of the disadvantages of consuming foods rich in sugar and salt. - Expose the risks of eating excessive amounts of red and processed meat. - Teach how to identify processed and ultra-processed foods. - Acquire knowledge to interpret nutrition labels.	- This session will expose the harmful effects of over-consumption of processed and ultra-processed foods. NOVA classification will be presented to identify them. We will also give the alternatives to reduce the consumption of red and ultra-processed meat. Finally, we will teach about the interpretation of nutrition labels.	- As a final activity, some participants will read and interpret the label of a nutritional product.
**8. Let’s plan our weekly food purchase!**	1 h	- Teach how to plan the food purchasing on a daily or weekly basis in a healthy way. - Apply the knowledge acquired during the previous session. - Propose healthy options for each food group. - Emphasize the purchase of fresh, seasonal and local food.	- This theoretical-practical session will teach about the planning of buying healthy food on a daily or weekly basis. Healthy options for each food group will be presented emphasizing the purchase of fresh, seasonal and local food.	- Prepare a grocery list conjointly.
**9. How to plan a balanced menu?**	1 h	- Explain how to plan a culturally adapted healthy menu. - Encourage the splitting of meals by clarifying their structure. - Practice making a healthy plate. - Apply the knowledge acquired during the previous sessions.	- This session will encourage the establishment of a meal schedule, following the healthy plate method (½ vegetables, ¼ starch and ¼ protein) and for the snack, we will recommend preparing healthy snacks based on fruit, vegetables, pulses and nuts. Finally, some ideas will also be proposed to combine all the meals respecting the portion and frequency of consumption of different foods.	- As a final activity, a 3–5 days menu will be prepared jointly. At the end of the session, a qualitative weekly menu will also be provided.
**10. Photovoice**	1 h	- Recognize the representative dishes of Pakistani cuisine. - Understand the meaning of food for the Pakistani community.	- In this last session, the participating women will bring a photo of a healthy dish or snack made with ingredients that have a very important meaning for them. There will be an exhibition of the images, so that each participant will share the meaning of their dish with their fellows, explaining the modifications they have made to convert their dish into a healthy plate.	- In this session, the participants will be allowed to come accompanied by their family and friends. They will present them their plates as well.
